# Is life course socioeconomic position associated with current self-rated health and happiness: results of World Values Survey 2018 for Türkiye

**DOI:** 10.1186/s12889-026-28978-7

**Published:** 2026-08-18

**Authors:** Isil Ergin, Asli Ata Teneler, Aliye Mandiracioglu, Handegul Caliskan Cebi

**Affiliations:** Faculty of Medicine, Department of Public Health, Ege University, Izmir, Türkiye

**Keywords:** Life course, Socioeconomic position, Self-rated health, Happiness, World Values Survey

## Abstract

**Background:**

Health and happiness are fundamental components of well-being, shaped by socioeconomic determinants across the life course. This study investigates the association of life course socioeconomic position (SEP)-encompassing childhood/origin SEP, current adulthood SEP and intergenerational social mobility-, with current self-rated health (SRH) and happiness in the Turkish population.

**Methods:**

Using cross-sectional data from the 7th wave of the World Values Survey for Türkiye (*n* = 1694), sex-stratified analysis were conducted. Age, educational attainment, income level, and social class were assessed for participants and their parents. These direct indicators of childhood and adult socioeconomic position were used to derive intergenerational social mobility measures. The primary outcome variables were self-rated health (SRH) and happiness. Bivariate chi-square tests and multivariate logistic regression models were utilized to examine bivariate and adjusted relationships, respectively.

**Results:**

In bivariate analyses, childhood and adult educational indicators were significantly associated with both SRH and happiness. However, in multivariate regression models adjusted for income and class components, the independent effect of education was largely attenuated. For poor SRH, the strongest independent determinant was current low income among women (OR = 2.93, 95%CI: 1.30–6.64), whereas downward income mobility was the key predictor among men (OR = 2.77, 95% CI: 1.84–4.18). For unhappiness, multivariate models revealed that women in the lowest current income tier had markedly higher odds (OR = 7.26, 95%CI: 2.16–24.43). For men, belonging to the lowest current social class was the strongest determinant of unhappiness (OR = 3.12, 95% CI: 1.34–7.24), followed by downward income mobility (OR = 1.74, CI: 1.07–2.83).

**Conclusion:**

Using a life-course approach, this study demonstrates that adult income and social class exert a primary influence on health and happiness, overshadowing the standalone effect of education. The observed life-course trajectories are deeply gendered. Interventions addressing health inequalities must prioritize reducing current income inequalities and removing structural gender barriers throughout life.

## Background

Biological, behavioral, and psychosocial processes along with their relevant socioeconomic determinants across the life span or across generations, influence disease risk [19]. The life course approach has been defined as a powerful framework for unravelling the role of socioeconomic inequalities throughout the life span for morbidity and mortality [28]. Key indicators of social status (such as education or occupation) or measures of material resources (such as income or wealth) have frequently been used for this purpose. Multidimensional theoretical constructs as well as their change over time have been used to capture the full range of the concept [26, 47]. In this context, research has increasingly focused on social mobility, which is defined as attaining a higher or lower socioeconomic position (SEP) relative to one's parents. Consequently, childhood SEP, adulthood SEP, and intergenerational social mobility have emerged as key explanatory variables influencing both health and happiness [1, 15]. Social mobility refers to change in an individual’s SEP, either in relation to their parents (inter-generational mobility) or throughout their lifetime (intra-generational mobility) [35].

Self-rated health (SRH) was frequently used as a summary measure of the individual’s perceptions of their overall health. Studies have consistently shown it to be a powerful predictor of clinical outcome and mortality [9, 13]. At the crossroads of culture and biology, the validity of SRH across different socioeconomic groups has been debated, but studies have shown it to be a strong predictor of subsequent mortality in all socioeconomic groups [25]. Happiness, the emotional dimension of subjective wellbeing, is specifically sensitive to socio-economic fluctuations. It captures the emotional quality of daily life. This distinguishes it from life satisfaction, a long-term cognitive life evaluation, which is more prone to social desirability bias and cultural filters masking the direct psychological impact of material deprivation [14, 22]. Happiness is a complex and multidimensional concept and is increasingly recognized as an important tool for guiding public policy and measuring the effectiveness of policy actions [30]. A systematic review of cross-cultural evidence demonstrates that while fundamental socioeconomic determinants of happiness show universal patterns, their specific influences can vary considerably across different economical, structural and cultural contexts [44]. Happiness and good health are fundamental to well-being and their intrinsic link necessitates a unified examination (Oshio and Kobayashi 2010). With this study, how the individual's SEP shapes happiness and health will be evaluated within a life course perspective. This analysis is particularly significant as it captures the cumulative effects of Türkiye’s rapid socioeconomic transition within this century, on individual life trajectories.

Among the many explanations, the effects of social mobility on health and happiness can be categorized into three main pathways: Harmful, beneficial and neutral [57]. Regarding the harmful pathway, the dissociative hypothesis suggests that socially mobile individuals may feel unaligned with both their origin and destination class. This chronic stress and sense of exclusion can subsequently lead to psychological stress and physical health problems. The frustrated achiever hypothesis asserts that “new entrants” are at a deficit than the original members and this can adversely affect their health. Downward mobility brings upon deteriorating effects on health, which is explained by the “falling from grace” thesis. For beneficial effects, the rags hypothesis implies the aspirational process to overcome the adversities. Thus, upward social mobility implies better SRH and happiness. The neutral theory relies on the individual’s integration to the new class. According to this acculturation hypothesis, the level of integration determines the health outcomes and the destination point has the most impact on health outcomes [57]. 

To understand social mobility in Türkiye, the country’s unique demographic and socioeconomic trajectory must also be considered. Türkiye’s population, which was 13.6 million during the early years of the Republic in 1927, doubled by 1960—a growth fueled by pronatalist policies and a Total Fertility Rate (TFR) of seven. The marked shift toward antinatalist policies in 1960’s and state-led development model, accelerated internal migration and urbanization, leading the TFR to decline to five by the 1970’s [20]. During this period, while traditional kinship ties and family inherited resources remained influential, education emerged as a primary lever for individual social mobility. The post 1980 era introduced a neoliberal market economy that fundamentally restructured social stratification. By the late 1980’s the TFR had dropped to three. The accelerated market integration after the 2000 s introduced a new landscape. In this new context, the transformative power of individual education was increasingly overshadowed by capital accumulation and instrumental social networks, which then became more potent drivers of social status. All this transition period profoundly altered the role of women; the traditional domestic centered model gave way to a market driven dual breadwinner household structure, where a women’s SEP began to decouple from her husband’s or her family of origin [38]. This transition has unfolded at an uneven pace marked by substantial regional differences, primarily a West–East divide. The persistent internal migration and a flow from rural to urban has resulted in an 89% urbanized population [50] hosting traditional and modernized social structures in contemporary Türkiye. Thus, in this compressed modernization of Türkiye, we explore how inherited status, marital alignment and individual achievement interact to shape health and happiness. Specifically we examine whether these dynamics- and their gendered patterns- diverge from those observed in Western societies where social structures evolved through more gradual and institutionalized transitions. Unlike the gradual mobility patterns of long-established market economies, the rapid transformation in Türkiye may empower or challenge psychological and physical health for different SEPs. This study aims to investigate the association of life course SEP with current SRH and happiness, in Türkiye. The explanatory variables were childhood and adulthood SEP, and intergenerational social mobility. For this purpose, data from the World Values Survey (WVS) 2018 for Türkiye was used.

## Methods

### Data

The national data of Türkiye from the most recent wave of the WVS (2017–2020) was used in this study [54]. WVS is an international research programme designed to assess social, political, economic, religious, and cultural values around the world [21]. This study utilizes the latest available WVS data from the Wave 7 global dataset (2017–22) as the subsequent 8th wave has not yet been released for public use [55]. This 7th wave of the survey began in mid-2017 and with a one-year gap due to the pandemic, was completed in December 2021. With a stratified multi-stage random sampling, Turkish data were collected in 2018 March–May via household face-to-face paper and pencil interviewing. The current time gap in this study does not diminish the representative power of the findings for intergenerational social mobility that unfolds over decades. This is particularly relevant given that Türkiye’s socioeconomic transformation has a century long trajectory that began with the founding of the Republic, gained a different momentum in the 1980 neoliberal pivot and accelerated in the 2000s.Weighted data were used for the analysis herein (*N* = 2415, men = 1207, women = 1208). A total of 721 individuals were excluded from analysis: missing data for SRH (*n* = 9) and happiness (*n* = 4) and those under 30 years of age (*n* = 708). The analysis included 1694 individuals aged ≥ 30 years (men = 826, women = 868). Among this WVS population, those above 65 years of age were only 1.8%. Thus, the current data predominantly represents the working age population who were born between the 1950 s and late 1980s.When considering their parents, this dataset effectively captures a comprehensive intergenerational perspective that spans several decades of Türkiye’s history. Access to the data was provided free of charge by registering and agreeing to the WVS non-redissemination data use license. This new version of the data includes a life course assessment of educational level, income and class with specific questions about the individual and their ancestors. Thus, the new version of the WVS was very valuable for the purposes of this study herein and the nationally representative nature also made it a reliable evaluation.

### Variables

In the study herein, two variables were specified as dependent: *SRH* and *happiness.* For SRH, participants were asked “All in all, how would you describe your state of health these days? Would you say it is…” and to choose from very good, good, fair, poor and very poor. This was dichotomised into very good/good and fair/poor/very poor, for analytical purposes and to ensure statistical power and interpretability. To measure happiness, respondents were asked “Taking all things together, would you say you are: Very happy, Happy, Quite happy, Not very happy, Not at all happy”. This was dichotomised as; very happy/happy/quite happy and not very happy/not at all happy.

The relationship of these variables with *age, education level, income and class* was determined. Respondents over the age of 30 were included in the analysis and grouped into five-year intervals. The age cut-off was chosen as 30, to represent established adulthood SEP. This ensured that the participants had transitioned from the developmental stage of emerging adulthood into a stage characterized by stabilized professional roles and completed educational pathway [32, 52].

For *education level*, “the highest educational level attained” was asked for self, spouse, mother and father. The educational groups were according to International Standard Classification of Education (ISCED) and the available groups in Turkish data were: ISCED 0: early childhood education or no education. ISCED 1:Primary education/ISCED 2:Lower secondary education/ISCED 3:Upper secondary education/ISCED 6:Bachelor or equivalent. To capture *intergenerational educational mobility*, two separate variables were constructed for this study: paternal and maternal educational mobility. The educational level of individual was positioned according to each parent. These were categorized as same (attaining the same level as the parent), upward mobility (attaining a higher level), downward mobility (attaining a lower level) for education.

For *current income*, the standardized household income variable provided in the official WVS Wave 7 dataset was used. The primary data was collected via a 10-point scale ("On this card is an income scale on which 1 indicates the lowest income group and 10 the highest income group in your country. We would like to know in what group your household is. Please, specify the appropriate number, counting all wages, salaries, pensions and other incomes that come in), we utilized the official WVS derived three-tier categorical income measure categorized as low (deciles 1–3), medium (deciles 4–7), and high (deciles 8–10). This standardized recoding was designed by the WVS team to ensure cross-national consistency and mitigate sparse-data issues in extreme income deciles. The level of income in childhood was not available among the questions. However, for intergenerational income mobility the question “Comparing your standard of living with your parents’ standard of living when they were about your age, would you say that you are better off, worse off or about the same?” was used and categorized as*:* same, upward income mobility(better off), downward income mobility(worse off).

For *current class*, respondents were asked “People sometimes describe themselves as belonging to the working class, the middle class, or the upper or lower class. Would you describe yourself as belonging one of them?” (WVS Survey-Q287) These four categories represented the subjective positioning and was predicted to be a good proxy for well-being. To assess intergenerational class mobility, a similar question/category was not available in the questionnaire for childhood class, so, we employed a comparative occupational status framework, given the limitations of available data. The social class of origin was determined using Q283 (When you were 14, to which of the following occupational groups did your father belong?), which identified the father's occupational group when the respondent was 14 years old. For comparing with the current occupational group with Q281 (To which of the following occupational groups do you belong?), there was a methodological challenge. In Q279 (Are you employed now or not?”), there were “homemakers" which were predominantly women. In line with sociological stratification studies in Türkiye, homemakers were classified based on the household-head approach [38]. Within this framework, the socioeconomic position of a household is defined by the occupation of the spouse which was available in WVS at Q282 (which of the following occupational groups does your spouse belong?). Thus to avoid the misclassification of homemakers, we adopted a spouse-based classification. This approach is a pragmatic proxy for household-level positioning. Both origin and destination statuses were categorized based on the occupational hierarchy in WVS Wave 7. Categories in the WVS questionnaire were hierarchically ordered to reflect a descending gradient of socio-economic prestige, skill requirements, required educational credentials, and administrative authority. This standardized structure of the dataset was preserved and used as follows:

- 1. Higher administrative (e.g., banker, big business executive, high government official)
- 2. Professional and technical (e.g., doctor, teacher, engineer, accountant, nurse)
- 3. Clerical (e.g., secretary, clerk, office manager, civil servant)
- 4. Sales (e.g., sales manager, shop owner, insurance agent)
- 5. Service (e.g., restaurant owner, police officer, barber, caretaker)
- 6. Skilled worker (e.g., foreman, motor mechanic, electrician, seamstress)
- 7. Semi-skilled worker (e.g., bricklayer, bus driver, carpenter, baker)
- 8. Unskilled worker (e.g., labourer, porter, cleaner)
- 9. Farm owner/Farm manager
- 10. Farm worker (e.g., farm labourer, tractor driver)

By comparing the respondent’s/household head’s occupational status against their father’s occupational status at age 14, three mobility trajectories were operationalized: same (remaining in the same occupational stratum), upward mobility (moving to a higher occupational tier than the father), downward mobility (moving to a lower occupational tier than the father).

### Statistical analysis

The relationship between SRH and happiness with age, education level (self, spouse, mother, father, and mobility), income (current and mobility) and class (current and mobility) were evaluated with Chi square analysis performed separately for two sexes. Standardized residuals were used to identify those specific cells making the greatest contribution to the chi-square test result. A standardized residual that is more than 1.96 or less than -1,96 indicated the group significantly differing from the rest. Following this, multilevel logistic regression was used to estimate differences for these two dependent variables in models including the independent variables determined to have significant relation with SRH and/or happiness. After checking for multicollinearity, the spouse educational level was eliminated from the models as it correlated highly with self**-**educational level for men (*r* = 0,748 *p* < 0,001) and women (*r* = 0,729 *p* < 0,001). For SRH; age, educational level(self), current income, income mobility, current class and class mobility were included in the Model for women while for men age, educational mobility(from father), current income, income mobility and current class were in the Model. For happiness, education level (self), educational mobility(from mother), current income level, income mobility current class and class mobility were in the Model for women while for men current income, income mobility and current class were in the Model. Odds ratios were calculated using the regression coefficients and their standard errors. The reference categories were the most advantageous socioeconomic groups identified in the univariate analysis and are presented for each variable in the Tables as *Ref*.

Additionally, a sensitivity analysis using bivariate chi-square tests was performed on a restricted subsample of employed women (*n* = 355) to evaluate whether the observed associations were driven by household-level proxy assignments for homemakers.

## Results

The characteristics of the study population for two sexes is presented in Table 1. The mean age was 45.1 ± 9.7 years and 51.2% were women. Those with an educational level of primary school or less were 55.3%. While 62.4% of women achieved a lower educational level (ISCED 0 and 1) compared to men (47.8%), higher frequency of men (17.3%) completed ISCED 6 level compared to women (11.3%). Low education level of mother (ISCED 0 and 1) was similar for women (64.4%) and for men (63.9%). For women, educational mobility downward (from father) was double (11.3%) the frequency for men (5.4%). Men were more frequently upwardly mobile than women (from father 60.6% vs 42.9% and from mother 67.9% vs 51.5%). The majority (75%) described their household income as medium. Those who described themselves as belonging to the middle class were 61.5%. Class mobility downward was more common for women (23.7%) than men (11.7%).

**Table 1:** The study population according to age, education, income and class

		**TOTAL**	**WOMEN**	**MEN**
AGE	Age (*n* = 1694)		**868**	826
	30–34	240 (14.2)	**112(12.9)**	128(15.5)
	35–44	550 (32.5)	**273(31.5)**	277(33.5)
	45–54	408 (24.1)	**213(24.5)**	195(23.6)
	55–64	466 (27.5)	**256(29.5)**	210(25.4)
	65 +	30 (1.8)	**14(1.6)**	16(1.9)
EDUCATION	Education level/self (*n* = 1687)		**864**	823
	ISCED 0	241 (14.3)	**167(19.3)**	74(9.0)
	ISCED 1	691(41.0)	**372(43.1)**	319(38.8)
	ISCED 2	187 (11.1)	**75(8.7)**	112(13.6)
	ISCED 3	328(19.4)	**152(17.6)**	176(21.4)
	ISCED 6	240(14.2)	**98(11.3)**	142(17.3)
	Education level/spouse (*n* = 1453)		**780**	673
	ISCED 0	123(8.5)	**39(5.0)**	84(12.5)
	ISCED 1	619 (42.6)	**315(40.4)**	304(45.2)
	ISCED 2	232(16.0)	**131(16.8)**	101(15.0)
	ISCED 3	339(23.3)	**203(26.0)**	136(20.2)
	ISCED 6	140 (9.6)	**92(11.8)**	48(7.1)
	Education level/mother (*n* = 1674)		**854**	820
	ISCED 0	676 (40.4)	**346(40.5)**	330(40.2)
	ISCED 1	733(43.8)	**375(43.9)**	358(43.7)
	ISCED 2	144 (8.6)	**67(7.8)**	77(9.4)
	ISCED 3 and 6	121 (7.2)	**66(7.7)**	55(6.7)
	Education level/father (*n* = 1674)		**854**	820
	ISCED 0	461 (27.5)	**239(28.0)**	222(27.1)
	ISCED 1	846 (50.5)	**438(51.3)**	408(49.8)
	ISCED 2	201 (12.0)	**89(10.4)**	112(13.7)
	ISCED 3 and 6	166 (9.9)	**88(10.3)**	78(9.5)
	Education mobility/from father (*n* = 1673)		**853**	820
	Same	670 (40.0)	**391(45,8)**	279(34,0)
	Upward	863(51,6)	**366(42,9)**	497(60,6)
	Downward	140 (8,4)	**96(11,3)**	44(5,4)
	Education mobility/from mother (*n* = 1672)		**853**	819
	Same	615 (36.8)	**369(43.3)**	246(30.0)
	Upward	995 (59.5)	**439(51.5)**	556(67.9)
	Downward	62 (3.7)	**45(5.3)**	17(2.1)
INCOME	Current Income (*n* = 1638)		**840**	798
	Low	250 (15.3)	**138(16.4)**	112(14.0)
	Medium	1229 (75.0)	**623(74.2)**	606(75.9)
	High	159 (9.7)	**79(9.4)**	80(10.0)
	Income mobility (*n* = 1663)		**854**	809
	Same	428(25.7)	**218(25.5)**	210(26.0)
	Upward	796 (47.9)	**398(46.6)**	398(49.2)
	Downward	439(26.4)	**238(27.9)**	201(24.8)
CLASS	Current class (*n* = 1650)		**846**	804
	Upper	18(1.1)	**11(1.3)**	7(0.9)
	Upper middle	466(28.2)	**220(26.0)**	246(30.6)
	Lower middle	550 (33.3)	**295(34.9)**	255(31.7)
	Working	483(29.3)	**246(29.1)**	237(29.5)
	Lower	133 (8.1)	**74(8.7)**	59(7.3)
	Class mobility (*n* = 1602)		**822**	780
	Same	610(38.1)	**297(36.1)**	313(40.1)
	Upward	706 (44.1)	**330(40.1)**	376(48.2)
	Downward	286(17.9)	**195(23.7)**	91(11.7)

The results for the relation of current and previous socioeconomic position/their change with SRH for two sexes are presented in Table 2. Among the study population, 30.2% perceived their health as fair/poor/very poor. SRH and happiness were moderately correlated (*r* = 0.43, *p* < 0.001). The prevalence of poor rated health status increased with age following an expected biological trend for both men and women. For women, regarding the educational indicators; the attainment of the woman herself, her spouse, and the parents (mother and father) were related with her current SRH. Being a highly educated woman (self), having a highly educated spouse and highly educated father/mother contributed most to the observed differences and low prevalence of poor rated health was significantly detected in these groups. However, women’s mother made an inverse contribution, and a high prevalence of poor rated health was observed among women with low educated mothers (Table 1). For men these variables showed no significant association with SRH. However, an upward educational mobility compared to the father contributed significantly to good health for men. For women, educational mobility (compared to mother or father) showed no significant relation for SRH. For both sexes, there was a significant association between current income and SRH. Low income and high-income groups contributed equally to this relation. While low income groups were more likely to report poor SRH, high income groups were less likely to do so. An income that has been worse off in comparison with early years of life was in relation with poor SRH, for both men and women. For the current class, the upper middle class declared poor SRH less frequently than other class groups for both sexes. Women who remained in the same class profile compared with their early years of life were less likely to report poor health than those who moved up or down (Table 2).

**Table 2:** The chi-square analysis results for the relation of current, origin SEP/their change for the study population with SRH for two sexes, WVS 2021, Türkiye (*N* = 1694)

		WOMEN			MEN		
		Healthy n(%)	Unhealthy n(%)	^χ2^ *p*	Healthy n(%)	Unhealthy n(%)	^χ2^ *p*
AGE	Age (*n* = 1694)			28,623 ** < 0.001**			18.248 **0.001**
	30–34	89(79.5)	23(20.5)		102(79.7)	26(20.3)	
	35–44	192(70.3)	81(29.7)		216(78.0)	61(22.0)	
	45–54	141(66.2)	72(33.8)		135(69.2)	60(30.8)	
	55–64	162(63.3)	94(36.7)		132(62.9)	78(37.1)	
	65 +	2(14.3)	12(85.7)		11(68.8)	5(31.3)	
EDUCATION	Education level/self (*n* = 1687)			22.165 ** < 0.001**			8.999 0.610
	ISCED 0	103(61.7)	64(38.3)		48(64.9)	26(35.1)	
	ISCED 1	236(63.4)	136(36.6)		221(69.3)	98(30.7)	
	ISCED 2	62(82.7)	13(17.3)		83(74.1)	39(34.8)	
	ISCED 3	102(67.1)	50(32.9)		127(72.2)	49(27.8)	
	ISCED 6	80(81.6)	18(18.4)		115(81.0)	27(19.0)	
	Education level/spouse (*n* = 1453)			13.121 **0.011**			5.638 0.228
	ISCED 0	21(53.8)	18(46.2)		58(69.0)	26(31.0)	
	ISCED 1	202(64.1)	113(35.9)		210(69.1)	94(30.9)	
	ISCED 2	87(66.4)	44(33.6)		70(69.3)	31(30.7)	
	ISCED 3	138(68.0)	65(32.0)		97(71.3)	39(28.7)	
	ISCED 6	75(81.5)	17(18.5)		41(85.4)	7(14.6)	
	Education level/mother (*n* = 1674)			11.463 **0.009**			7.078 0.069
	ISCED 0	213(61.6)	133(38.4)		222(67.3)	108(32.7)	
	ISCED 1	266(70.9)	109(29.1)		270(75.4)	88(24.6)	
	ISCED 2	46(68.7)	21(31.3)		59(76.6)	18(23.4)	
	ISCED 3 and 6	52(78.8)	14(21.2)		42(76.4)	13(23.6)	
	Education level/father (*n* = 1674)			9.895 **0.019**			3.306 0.347
	ISCED 0	149(62.3)	90(37.7)		156(70.3)	66(29.7)	
	ISCED 1	297(67.8)	141(32.2)		294(72.1)	114(27.9)	
	ISCED 2	60(67.4)	29(32.6)		80(71.4)	32(28.6)	
	ISCED 3 and 6	71(80.7)	17(19.3)		63(80.8)	15(19.2)	
	Education mobility/from father (*n* = 1673)			3.778 0.151			6.261 **0.044**
	Same	252(64,5)	139(35,5)		189(67,7)	90(32,3)	
	Upward	260(71,0)	106(29,0)		375(75,5)	122(24,5)	
	Downward	64(66,7)	32(33,3)		29(65,9)	15(34,1)	
	Education mobility/from mother (*n* = 1672)			1.502 0.472			5.301 0.071
	Same	244(66.1)	125(33.9)		165(67.1)	81(32.9)	
	Upward	304(69.2)	135(30.8)		413(74.3)	143(25.7)	
	Downward	28(62.2)	17(37.8)		14(82.4)	3(17.6)	
INCOME	Current Income (*n* = 1638)			20.549 ** < 0.001**			17.247 ** < 0.001**
	Low	76(55.1)	62(44.9)		69(61.6)	43(38.4)	
	Medium	424(6.1)	199(31.9)		438(72.3)	168(27.7)	
	High	67(84.8)	12(15.2)		71	9(11.3)	
	Income mobility (*n* = 1663)			6.959 **0.031**			24.349 ** < 0.001**
	Same	150(68.8)	68(31.2)		143(68.1)	67(31.9)	
	Upward	284(71.4)	114(28.6)		320(80.4)	78(19.6)	
	Downward	146(61.3)	92(38.7)		126(62.7)	75(37.3)	
CLASS	Current class (*n* = 1650)			37.697 ** < 0.001**			23.009 ** < 0.001**
	Upper	10(90.9)	1(9.1)		7(100)	0(0)	
	Upper middle	172(78.2)	48(21.8)		200(81.3)	46(18.7)	
	Lower middle	168(56.9)	127(43.1)		174(68.2)	81(31.8)	
	Working	180(73.2)	66(26.8)		167(70.5)	70(29.5)	
	Lower	41(55.4)	33(44.6)		33(55.9)	26(44.1)	
	Class mobility (*n* = 1602)			6.540 **0.038**			3.237 0.198
	Same	216(72.7)	81(27.3)		222(70.9)	91(29.1)	
	Upward	219(66.4)	111(33.6)		289(76.9)	87(23.1)	
	Downward	121(62.1)	74(37.9)		66(72.5)	25(27.5)	

Note: Bold values indicate statistically significant results

The results for the relationship between current and previous socioeconomic position/their change with happiness for two sexes are presented in Table 3. Among the study group, those feeling not very happy/not at all happy were 14.5%. The happiness status of women was related to the educational level of the woman and her spouse. Those in the ISCED 3 group (upper secondary education) were more often unhappy than the others, both for themselves and for their spouses. While women who did not move up or down in educational level were less likely to be unhappy, those who moved up were more likely to be unhappy. Regarding income, women with low-income were more frequently unhappy opposite to women with high income. Unhappiness was less prevalent among those who had moved upward for a better standard of living and more prevalent among the worse off. For men, age and education indicators were not related with happiness status. The lowest income group of men had the highest prevalence of unhappiness, and those who had achieved a lower income status were the most likely to be unhappy. Men who described themselves as lower class were more likely to be unhappy (Table 3).

**Table 3:** The Chi-square analysis results for the relationship between current and origin SEP/their change for the study population with feeling of happiness for two sexes, WVS 2021, Türkiye (*N* = 1694)

		WOMEN			MEN		
		Happy n(%)	Unhappy n(%)	^χ2^ *p*	Happy n(%)	Unhappy n(%)	^χ2^ *p*
AGE	Age			1.836 0.766			4.622 0.328
	30–34	96(85.7)	16(14.3)		113(88.3)	15(11.7)	
	35–44	230(84.2)	43(15.8)		237(85.6)	40(14.4)	
	45–54	179(84.0)	34(16.0)		159(81.5)	36(18.5)	
	55–64	223(87.1)	33(12.9)		183(87.1)	27(12.9)	
	65 +	13(92.9)	1(7.1)		15(93.8)	1(6.3)	
EDUCATION	Education level/self			13.100 **0.011**			2.492 0.646
	ISCED 0	150(89.8)	17(10.2)		65(87.8)	9(12.2)	
	ISCED 1	313(84.1)	59(15.9)		273(85.6)	46(14.4)	
	ISCED 2	70(93.3)	5(6.7)		95(84.8)	17(15.2)	
	ISCED 3	119(78.3)	33(21.7)		146(83.0)	30(17.0)	
	ISCED 6	85(86.7)	13(13.3)		126(88.7)	16(11.3)	
	Education level/spouse			10.038 **0.040**			0.727 0.948
	ISCED 0	37(94.6)	2(5.1)		72(85.7)	12(14.3)	
	ISCED 1	273(86.7)	42(13.3)		259(85.2)	45(14.8)	
	ISCED 2	117(89.3)	14(10.7)		85(84.2)	16(15.8)	
	ISCED 3	162(79.8)	41(20.2)		112(82.4)	24(17.6)	
	ISCED 6	79(85.9)	13(14.1)		41(85.4)	7(14.6)	
	Education level/mother			5.888 0.117			7.108 0.069
	ISCED 0	292(84.4)	54(15.6)		271(82.1)	59(17.9)	
	ISCED 1	332(88.5)	43(11.5)		313(87.4)	45(12.6)	
	ISCED 2	54(80.6)	13(19.4)		71(92.2)	6(7.8)	
	ISCED 3 and 6	53(80.3)	13(19.7)		48(87.3)	7(12.7)	
	Education level/father			0.210 0.976			4.214 0.239
	ISCED 0	206(86.2)	33(13.8)		185(83.3)	37(16.7)	
	ISCED 1	374(85.4)	64(14.6)		348(85.3)	60(14.7)	
	ISCED 2	75(84.3)	14(15.7)		102(91.1)	10(8.9)	
	ISCED 3 and 6	75(85.2)	13(14.8)		69(88.5)	9(11.5)	
	Education mobility/from father			3.144 0.208			0.309 0.857
	Same	336(85.9)	55(14.1)		242(86.7)	37(13.3)	
	Upward	306(83.6)	60(16.4)		424(85.3)	73(14.7)	
	Downward	87(90.6)	9(9.4)		3(86.4)	6(13.6)	
	Education mobility/from mother			6.855 **0.032**			1.121 0.571
	Same	329(89.2)	40(10.8)		212(86.2)	34(13.8)	
	Upward	363(82.7)	76(17.3)		474(85.3)	82(14.7)	
	Downward	38(84.4)	7(15.6)		16(94.1)	1(5.9)	
INCOME	Current Income			26.969 ** < 0.001**			9.904 **0.007**
	Low	99(71.7)	39(28.3)		85(75.9)	27(24.1)	
	Medium	541(86.8)	82(13.2		524(86.5)	82(13.5)	
	High	75(94.9)	4(5.1)		72(90.0)	8(10.0)	
	Income mobility			12.175 **0.002**			6.476 **0.039**
	Downward	189(79.4)	49(20.6)		163(81.1)	38(18.9)	
	Upward	356(89.4)	42(10.6)		353(88.7)	45(11.3)	
	Same	185(84.9)	33(15.1)		179(85.2)	31(14.8)	
CLASS	Current class			14.058 **0.007**			24.798 ** < 0.001**
	Upper	11(100)	0(0)		7(100.0)	0(0)	
	Upper middle	195(88.6)	25(11.4)		218(88.6)	28(11.4)	
	Lower middle	246(83.4)	49(16.6)		216(84.7)	39(15.3)	
	Working	214(87.0)	32(13.0)		207(87.3)	30(12.7)	
	Lower	54(73.0)	20(27.0)		38(64.4)	21(35.6)	
	Class mobility			8.031 **0.018**			0.064 0.968
	Same	268(90.2)	29(9.8)		267(85.3)	46(14.7)	
	Upward	275(83.3)	55(16.7)		322(85.6)	54(14.4)	
	Downward	161(82.6)	34(17.4)		77(84.6)	14(15.4)	

Note: Bold values indicate statistically significant results

Table 4 summarizes the results of our logistic regression models for fair/poor health and unhappiness. Predictors were selected for each gender and outcome based on preliminary bivariate analyses. The model showed that fair/poor health was almost three times higher in women aged 55 and over than in younger ones (OR: 2.83 95%CI; 1.56–5.15); and two times higher in older men (OR: 2.02 95%CI; 1.12–3.62). The risk of being unhealthy was 2.93 times higher for women (OR: 2.93, 95% CI: 1.30–6.64) and 2.62 (OR: 2.62, 95% CI: 1.03–6.64) times higher for men (*p* < 0.05) in the low-income group compared to the high-income group. For women, a downward deterioration in income mobility is associated with a 1.53-fold increased risk of poor health (OR: 1.53, 95% CI: 1.04–2.26). Women in the lower/middle class were found to be 1.97 times less healthy (OR: 1.97, 95% CI: 1.28–3.04) than women in the other classes. In addition, men in the lower class were 2.20 (OR: 2.20, 95%CI: 1.02–4.71) times less healthy than those in the other classes. In terms of class, mobility downwards (OR:2.04, 95%CI: 1.33–3.14) and upwards (OR:1.55, 95%CI: 1.07–2.26) increased the risk of being unhealthy for women (*p* < 0.05).

**Table 4:** The results of the logistic regression analysis for fair/poor health and unhappiness for two sexes, WVS 2021, Türkiye^a^

	FAIR/POOR HEALTH		UNHAPPINESS	
	WOMEN	MEN	WOMEN	MEN
	OR (%95CI)	OR (%95CI)	OR (%95CI)	OR(%95CI)
Age				
30–34 (ref)	1.00	1.00	-	-
35–44	1.64(0.92–2.92)	0.92 (0.52–1.64)	-	-
45–54	1.92(1.06–3.49)*	1.76 (0.98–3.16)	-	-
55 +	2.83(1.56–5.15)**	2.02 (1.12–3.62)*	-	-
Education level/self				
ISCED 0	1.35(0.68–2.71)	-	0.549(0.18–1.64)	-
ISCED 1	1.63(0.89–3.00)	-	0.881(0.41–1.91)	-
ISCED 2	0.76(0.32–1.78)	-	0.341(0.10–1.15)	-
ISCED 3	1.81(0.94–3.50)	-	1.859(0.88–3.93)	-
ISCED 6(ref)	1.00	-	1.00	-
Educational mobility/from father				
Same (ref)	-	1,00	-	-
Upward	-	0,97 (0,68–1,40)	-	-
Downward	-	1,40 (0,67–2,93)	-	-
Educational mobility/from mother				
Same (ref)	-	-	1.00	-
Upward	-	-	1.68(0.96–2.96)	-
Downward	-	-	2.67(0.98–7.26)	-
Current income				
Low	2,93(1,30–6,64)*	2,62 (1,03–6,64)*	7.26(2.16–24.43)*	1.66(0.59–4.68)
Medium	1,82 (0,92–3,60)	1,86 (0,86–4,01)	2.65(0.89–7.80)	1.30(0.56–3.04)
High (ref)	1,00	1,00	1.00	
Income mobility				
Upward (ref)	1,00	1.00	1.00	1.00
Same	1,35(0,90–2,02)	2,10 (1,39–3,17)**	1.46(0.85–2.53)	1.26(0.76–2.11)
Downward	1,53(1,04–2,26)*	2,77 (1,84–4,18)**	1.89(1.12–3.19)*	1.74(1.07–2.83)*
Current class				
Upper/Upper middle (ref)	1,00	1,00	1.00	1.00
Lower middle	1,97(1,28–3,04)*	1,54 (0,98–2,41)	1.40(0,78–2,51)	1,34(0,77–2,33)
Working	0,87 (0,53–1,42	1,57 (0,97–2,54)	1,02(0,52–1,99)	1,09(0,60–2,01)
Lower	1,46 (0,71–3,01)	2,20 (1,02–4,71)*	1,69(0,69–4,14)	3,12(1.34–7.24)*
Class Mobility				
Same (ref)	1,00	-	1.00	-
Upward	1,55 (1,07–2,26)*	-	1,87(1,11–3,15)*	-
Downward	2,04 (1,33–3,14)*	-	2,14(1,19–3,83)*	-

^a^Predictors were selected for each gender and outcome based on preliminary bivariate analyses

Note: Bold values indicate statistically significant results

^*^p < 0.05

^**^p < 0.001

The risk of being unhappy increased 7.26 times (OR:7.26, 95%CI: 2.16–24.43) for women in the low income group compared to those in the higher income group. Downward mobility in terms of income makes women 1.89 times (OR: 1.89, 95%CI: 1.12–3.19) and men 1.74 times (OR: 1.74, 95%CI: 1.07–2.83) more likely to be unhappy. It was found that men in the lowest class were 3.12 (OR: 3.12, 95%CI: (1.34–7.24) times unhappier than men in the other classes. The downward (OR: 2.14, 95%CI: 1.19–3.83) and upward (OR: 1.87, 95%CI: 1.11–3.15) class mobility led to an increased risk of being unhappy in women (*p* < 0.05) (Table 4).

### Sensitivity analysis

To test whether the observed mobility-health (and happiness) dynamics among women were driven by household-level proxy assignment, a sensitivity analysis was conducted restricting the sample to employed women (*n* = 355). In this restricted sub-sample, the association between intergenerational class mobility and subjective health (χ^2^ = 1.347 and *p* = 0.510) and happiness (χ^2^ = 3.945 and *p* = 0.139) lost its statistical significance, mirroring the pattern observed among men.

## Discussion

In this study, using the Türkiye data from the 7th WVS, the SEP factors affecting SRH and happiness in the community and their intergenerational change were analyzed separately for men and women. The stark divergence in educational outcomes is particularly noteworthy given that both sexes shared nearly identical intergenerational starting points, as evidenced by the similar rates of low maternal education (approx. 64% for both). This indicates that family background alone does not fully account for the observed gender gap. Instead, it points toward the potential influence of gender-differentiated social dynamics that favor male advancement while hindering female educational attainment. While men predominantly followed same or upward mobility trajectories, women exhibited a notable vulnerability to downward socioeconomic shifts. These results highlight that educational attainment and class stability remain deeply gendered in Türkiye, necessitating policies that address the specific structural hurdles faced by women across their life course.

Our findings highlight a multifaceted interplay between socio-economic factors and subjective well-being indicators. The strongest determinants of poor health were current low-income status for women and downward income mobility for men. Being in the lower-middle class (for women) and lower class (for men) increased poor SRH and downward mobility negatively impacted health in both sexes. For happiness, current low-income for women and current lower class for men were the main determinants. This study also showed that for women, downward mobility of income and class and, for men downward mobility of income significantly influenced happiness. Neither achieved level of education nor its mobility within generations was no longer significant in the regression model when adjusted for other variables.

### Self-rated health

In this study, bivariate analysis revealed that higher parental education had a significant health-promoting effect for women, though this was not observed for men. While this initial finding highlights a noteworthy gender specific pattern, the subsequent loss of significance in the multivariate model-after adjusting for income and class- suggest that the role of parental education on health was mediated by these socioeconomic resources. Thus, the impact of education was largely realized by the material and structural advantages it facilitates. In a study conducted in the United States by Assari et. al, the parent's higher education level was found to be associated with better health and happiness, and its positive effects were found to be more pronounced for women, similar to our study. There is a need to understand the social, economic, and structural mechanisms, as well as the behavioural mechanisms that explain why parental education loses some of its effects for men compared to women. One underlying mechanism may be the differential impact of parental resources due to different investments in sons and daughters [1]. According to resource substitution theory, the beneficial effect of education on health is greater for people who have fewer alternative resources [41]. Because one's own educational attainment is highly structured by the educational attainment of one's parents, individuals from disadvantaged family backgrounds may themselves be disadvantaged. Those with few alternative resources are more dependent on education for health than those with more resources because resources are interchangeable to improve health. This theory predicts that people with low educated parents will benefit more from education in terms of health than those whose parents are well educated. In other words, it can be said that a person's level of education reduces the negative impact of low parental education level on health. Individuals who can improve their educational attainment despite low parental education can interrupt or at least weaken the link between disadvantaged family background and poor health. Education plays a crucial role in this process because education is a resource and the human capital developed through education helps people to produce other resources. By developing competence at many levels, education gives people the ability and motivation to shape and control their lives. A high level of education increases the sense of personal control,it can support the adoption of healthier lifestyles by giving people the belief that they can control or effectively change their environment. Compared with people who feel powerless, people with a sense of personal control are more likely to make healthy lifestyle choices, such as quitting smoking, exercising, and consuming moderate amounts of alcohol. These people appear to have better self-assessment of health and lower mortality rates. Education also gives people access to high-paying skilled jobs [42]. Educational attainment reflects both the economic constraints of parents and class-specific behavior during upbringing and is reported to be a very important determinant of class position in adult life. It has been found that highly educated individuals have better access to resources, experience less stress, make better decisions, and exhibit less risky health behaviors [16]. In our findings, in addition to one's own and parents' education, the spouses education level was also relevant for women. This suggests the persistent influence of partriarchal family structures in contemporary Türkiye, tying the women’s health outcomes to her spouses/parents status. However, the loss of significance in further analysis, reveals that this influence is operationalized through material pathways. Regarding the neoliberal economic restructuring of social stratification in Türkiye, education promotes health to the extent that it can be converted into high income and material resources. This transition is further amplified by the rise of the dual-breadwinner model in Türkiye. As women increasingly participate in the labor market, household health outcomes are no longer dictated by a single provider, but are shaped by the combined educational and economic capital of both partners. This result challenge the traditional view of parental education as a direct and lifelong health protector. In today’s economic landscape, this link is more about the successful translation of educational capital into economic resources. The ineffectual role of educational attainment in shaping men’s health suggests other structural drivers are at play.

Current results indicate a link between lower income and its downward mobility with SRH for both genders. Thus far, several studies have reported that income is strongly correlated with an individual's health [8, 14, 18, 46]. Despite this, only a few studies have investigated the role of intergenerational income mobility in determining health outcomes. A positive association between county-level income mobility and life expectancy was found in an ecological study for the United States [48]. Another study finds that upward income mobility is associated with better mental health, but also with higher rates of metabolic syndrome [33]. A Japanese quasi-experimental study suggests that, contrary to expectations, upward income mobility can have a negative effect on people's health [37]. According to a longitudinal study, downward income mobility was associated with increased cardiovascular risk, but only among people with poor initial health [6]. As suggested by the “falling from grace” thesis, the struggle to adapt and perception of failure has negative emotional consequences with lasting scars leaving addictive behaviors, anxiety, depression and chronic diseases [57]. In our study, while the men’s health is promoted by upward income mobility, women’s health is threatened by downward shifts. This suggests that health outcomes for men are tied to their success in the competitive labor market and whereas for women health is more sensitive to the loss of material resources. According to our results income is the strongest determinant of women's health, and the magnitude of the income effect was larger for women than men. This reinforces the argument about their heightened sensitivity to material resources. Remarkably, the risk associated with low income surpasses even the impact of biological aging. In contemporary Türkiye, income does not only complement education, but it effectively replaces it as the primary mechanism which women can access quality healthcare, better living conditions and social security. In the Turkish context, failure to strengthen social protection mechanisms risks health and happiness to be a market dependent privilege. This effect is more pronounced in women than in men. Over the last two decades, neoliberal healthcare reforms in Türkiye have redefined individuals as customers within a marketized system, thereby eroding the protective role of education. Consequently, access to health services became dependent on material resources. The right to health is increasingly replaced by individualized responsibility, leaving vulnerable populations -particularly women without stable market power-exposed to the economic shocks in an unstable economy [24].

In our study, being in the upper middle class was found to be the most advantageous baseline for health outcomes among women and men. These results are in accord with recent studies [40, 45, 49] indicating that self-reported socioeconomic position associate with self-reported health. A county-level study in the United States revealed that across all counties, moving from the lowest to highest levels of social status was associated with a reduction in the longevity gap by 1.4 years among men and 1.1 years among women [48]. Another similar example from Tehran reported that SRH was found to be poorer among participants with lower socioeconomic position [34]. Generally, people in lower socioeconomic groups are at greater risk of poor health, have higher rates of illness, disability and death [45]. The lower the social class, the higher the level of chronic psychological stress, the fewer the resources to control their environment and therefore experience uncertainties, helplessness, and lack of autonomy [12, 39, 51]. On the contrary, upper class individuals have more social, financial, and educational resources at their disposal allowing them to feel socially valued and in control of their lives. Chronic deprivation leads to subjective uncertainty about survival, which triggers the threat response and associated chronic activation of the sympathetic system and overproduction of inflammatory cytokines [31, 36]. This clarification broadly supports the Australian Institute of Health and Welfare report that compared to people living in the highest socioeconomic areas, those living in the lowest socio-demographic groups are more likely to suffer from obesity, physical inactivity and uncontrolled hypertension. They also have a higher burden of type 2 diabetes, lung cancer, coronary heart disease, stroke and dementia [2]. Our results suggested that while downward income mobility universally harms health for two sexes, social class mobility presents a unique gendered pattern. For women, any shift in social class-whether upward or downward-is associated with poorer health outcomes. The effect of socioeconomic position on health inequalities is cumulative [29]. With chronically advantaged (or disadvantaged) people having better (or worse) health, and social mobility moderating this cumulative effect [40]. Ironically, although high levels of upward social mobility are politically and philosophically preferable, the status-based identity ambiguity triggered by social mobility increases the person's allostatic load, triggering the body's stress response and reducing subjective well-being [43]. In a 2017 article by Destin et al. [10] in which they tried to understand the effects of social mobility on individuals' mental health and subjective well-being,Social class transitions, as a result of inherent uncertainty, exacerbate rather than combat inequality, particularly in terms of health and well-being outcomes [27]. The dissociative hypothesis revealing adverse health effects by any form of social mobility align with our findings. The erosion of the social capital is an important driver in this context [57] and for women in Türkiye, moving upward or downward from a social strata may disrupt their established social capital and support networks, leading to inconsistencies in their status. Other possible explanations are the stress of adapting to a new social class, the threat of exclusion, discrimination, and feeling like an imposter, as well as negative health consequences due to social isolation because of less interaction with their social class of origin. In other words, their social identities must be radically rewritten, and these rewriting breeds uncertainty and chronic stress [43]. As people move upward or downward, their understanding of their position in society can disintegrate. This can lead to high levels of status insecurity, which can be detrimental to motivation and well-being [10]. Even it is not yet clear why women tend to be more affected than men, the literature suggests that women may have poorer health due to weaker labour market attachment, lower social class, less civic participation, and the dual burden of paid work and household responsibilities [5, 7]. The sensitivity analysis also revealed that when the sample was restricted to employed women, the health and happiness associated with class mobility completely disappeared, mirroring the pattern observed among men. This contrast highlights that the mobility-health (and happiness) relationship in the broader female population is predominantly shaped by household/marital social destination rather than individual occupational mobility. Consequently, health inequalities among women appear to be more sensitive to household material living standards than to individual labor market trajectories.

However, a major conceptual limitation concerns the interpretation of female mobility trajectories. Because homemakers' social positions were operationalized via their spouses' occupations (513 women comprising 59.1% of female participants), women’s intergenerational mobility in this study partially reflects marital sorting and gendered household arrangements rather than autonomous occupational mobility. While this conventional approach captures household-level resource access in contexts with low female labor force participation, it highlights a broader methodological gap and a theoretical shortcoming: the persistent methodological dependency on defining women’s social standing through male figures. There is an urgent need for research focused specifically on conceptualizing and measuring the class positions of homemakers. Future studies should develop and validate multi-dimensional stratification tools that incorporate women's individual educational capital, intra-household resource allocation, and autonomous decision-making power to better capture female social positioning.

### Happiness

For women, income is the most potent predictor of happiness. Downward income mobility is also an important driver of unhappiness, for both sexes. Our findings were supported by several studies. According to the results of an analysis of 67 countries by WVS, gross domestic product per capita was found to be positively associated with happiness (Kim 2018). Another study showed a strong positive relationship between household SES and happiness, as well as between subjective socioeconomic position and happiness [4]. Our results are also consistent with those of the Taiwan Social Change Survey, which found that for women, income and changes in income have a significant impact on people's happiness. This study also shows that gender, age, education level, marital status, and occupation also significantly influence happiness. Women are happier than men, and middle-aged people are less happy than young and old people (Tsui 2014). According to the results of Türkiye 2018 life satisfaction survey,it has been stated that income, health and employment status were found to be important determinants of happiness and life satisfaction [23]. Downward income mobility poses risks for happiness too, which can be explained with the falling from grace hypothesis, signifying a reduction in cumulative material resources, the erosion in social capital and psychological support [57].

This study found that the upward or downward class mobility of women was destructive for happiness. The dissociative hypothesis suggesting any form of mobility to have detrimental effects is parallel with this finding [57]. Another explanation may be the Easterlin paradox. As Easterlin [11] points out, it is the relative rather than the absolute that is important for a person’s happiness, since happiness depends directly on one’s own income and inversely on the income of others. In fact, social comparison dominates the well-being of individuals and their decisions, which means that individuals in a society have their own social priorities and compare their level of welfare with that of others [14]*.* The "Easterlin paradox" states that income growth does not necessarily lead to an increase in self-rated well-being, as material wealth accumulates, it ceases to promote further improvements in well-being after a certain threshold [56]. From a sociological perspective, the deprivation theory introduced by Runciman states that the feelings of individuals are determined by their reference groups. Thus, they may feel relatively deprived if they observe that others have a better socio-economic position than themselves [14]. For men, currently belonging to the lower class or having a downward class mobility was associated with a higher odds of unhappiness. This can be interpreted with their provider role and social prestige. In the Turkish context, men’s subjective well-being is often closely linked with their positioning in the labor market and their ability to maintain or exceed their father’s occupational status. The social class represents the broader sense of identity. Therefore, falling to a lower occupational tier likely triggers a sense of status anxiety and psychological distress that is more pronounced in men than in women.

### Strength and limitations

The use of a population-based, stratified sample and the rigorous validity checks of the WVS dataset, constitute the primary strengths of this study [21]. The current secondary data analysis has also benefited from the large number of respondents to represent the whole country.

As the main questionnaire did not include direct questions on income and class in childhood, the researchers created mobility variables using data on parental status. It was assumed that these data could be used instead of childhood data, as they asked about the income and class status of their parents when the respondents were children. This introduces the possibility of recall bias.

Another limitation relates to the operationalization of household income. To align with WVS standardized indicators and prevent small cell counts, income was analyzed using the official three-level categorized variable (low, medium, and high) derived from the original 10-point scale. While this aggregation enhances statistical stability and comparability, it inevitably compresses socioeconomic variability within tiers. Consequently, potential non-linear dynamics or subtle intra-category income disparities regarding health and happiness outcomes may be obscured. Future studies could benefit from analysing the raw 10-point scale directly where sample sizes per cell permit.

A structural limitation stems from the cross-generational comparability of occupational classifications within a rapidly transforming economy. While the standard WVS occupational hierarchy provides a reliable proxy for broad socio-economic gradients, intergenerational mobility measures may not fully capture the shifting social weight of agrarian strata over time. Particularly in contexts undergoing historical rural-to-urban migration, agricultural categories encompass heterogeneous positions—ranging from smallholder subsistence farming to capital-intensive agricultural management—that require granular data on land ownership size, irrigation access, and wage-labor employment. Consequently, parental agrarian status may reflect a more complex socio-economic reality than implied by a unified ordinal ranking. Building upon the analytical tradition initiated by scholars like Boratav [3], granular inquiries that systematically unearth rural class stratification remain vital for fully comprehending Türkiye's historical class transformations and their contemporary health and social consequences. Future mobility research in emerging economies should prioritize incorporating these refined agrarian stratification parameters alongside urban occupational models.

To assess the health status of respondents from Türkiye, the study herein used SRH based on self-reported health information. A single question asking individuals to rate their overall health on a scale from very good to very poor is one of the most used measures of self-reported health. There is general agreement that this simple global question is a useful summary of individuals' perceptions of their overall health status [13]. SRH is not only an expression of the absence of disease, but also provides an indication of the status of general health and wellbeing across physical, psychological and social dimensions [53]. Similarly, the use of a single question for happiness has also been detected to be a powerful indicator of psychological well-being. It is evaluative in nature, and broader in its time coverage. It is highly correlated with other measures of happiness [17].

Another limitation of this study is the use of happiness as the sole measure of subjective well-being, rather than a life satisfaction scale. There is an ongoing debate in the literature regarding these two constructs; while life satisfaction offers a cognitive and stable evaluation of one’s life, happiness captures more immediate emotional responses to life events. In our analysis of social mobility, we favored the affective sensitivity of happiness to capture the change in income or class position. However, we acknowledge that this choice may overlook the long-term cognitive adjustments individuals make, and future studies would benefit from incorporating both dimensions to provide a more holistic view. Moreover, the subjective evaluation of other key variables-such as SRH, perceived social class and income mobility- introduces potential for reporting or social desirability bias. Thus, our results should be interpreted with caution when generalizing to objective socioeconomic indicators or clinical health outcomes.

## Conclusion

Using a life-course approach, this study demonstrates that adult income and social class exert a primary influence on health and happiness, overshadowing the standalone effect of education. In Türkiye, the rapid transformation to a market economy has redefined the links between human capital and health, often making material gains more decisive than educational attainment. This market dependency and the lack of institutional buffers raises concerns on the positive health outcomes provided by the impact of education throughout life. This new era, would create profound vulnerability during periods of economic instability, leading to detrimental impacts on health and happiness. Policies to combat health inequalities need an intense focus on income inequalities throughout life. Failure to strengthen social protection mechanisms risks health and happiness to be a market dependent privilege. The observed life-course trajectories are deeply gendered. Interventions addressing health inequalities must prioritize reducing current income inequalities and removing structural gender barriers that facilitate male advancement while penalizing female social mobility throughout life.

## Data Availability

The data underlying the results presented in the study are available from https://www.worldvaluessurvey.org/WVSDocumentationWV7.jsp?COUNTRY=3460

## Abbreviations

SRH: Self-rated health
SEP: Socio-economic positions
WVS: World Values Survey
ISCED: International Standard Classification of Education
